# Including ‘seldom heard’ views in research: opportunities, challenges and recommendations from focus groups with British South Asian people with type 2 diabetes

**DOI:** 10.1186/s12874-020-01045-4

**Published:** 2020-06-15

**Authors:** Suman Prinjha, Nasima Miah, Ebrahim Ali, Andrew Farmer

**Affiliations:** 1 Nuffield Department of Primary Care Health Sciences, University of Oxford, Radcliffe Observatory Quarter, Woodstock Road, Oxford, OX2 6GG UK; 2 Centre for BME Health, Diabetes Research Centre, University of Leicester, Leicester General Hospital, Gwendolen Road, Leicester, LE5 4PW UK; 3 Bangladesh Youth & Cultural Shomiti (BYCS), 30-32 Biddulph Street, Leicester, LE2 1BF UK

**Keywords:** Focus groups, South Asian, Diabetes, Seldom heard, Qualitative methods, Reflexivity, Patient and public involvement

## Abstract

**Background:**

The inclusion of ‘seldom heard’ groups in health and social care research is increasingly seen as important on scientific, policy and ethical grounds. British South Asians, the largest minority ethnic group in the United Kingdom (UK), are under-represented in health research yet over-represented in the incidence of certain conditions such as type 2 diabetes. With the growing requirement of patient involvement in research and the inclusion of diverse populations, methodological guidance on how to include, engage and conduct research with UK South Asian populations is essential if services and interventions are to be relevant and impactful. However, such guidance for researchers is limited.

**Methods:**

The aim of the paper is to reflect on our experiences of conducting focus groups with UK South Asian communities with type 2 diabetes, which involved experienced community partners and researchers working closely together. We discuss the factors that aided the successful delivery of the project, the challenges that we encountered, how we dealt with these, and recommendations.

**Results:**

Our study suggests ways to involve and conduct focus groups with UK South Asian populations. Key considerations are categorised under four headings: co-working with community partners; linguistic competency; cultural competency and awareness; and reflexivity, power and acknowledgement. Having an experienced team of researchers and community experts – with the relevant linguistic and cultural competencies and different kinds of knowledge and skills – was key to the successful delivery of the study. Working collaboratively enabled us to recruit a diverse sample, to navigate the challenges of recruitment, to be present at every discussion which helped contribute to data richness, and to reflect on our own roles in the research process.

**Conclusions:**

Focus groups with UK South Asian communities can be a useful way of exploring new topics and involving seldom heard views. While a useful method, focus groups are only one way of exploring a research topic and provide an insight into context-specific attitudes and views. Future research should explore British South Asian participants’ views on how they would like to be involved in research, including new methods of collecting data and coproducing research.

## Background

The inclusion of ‘seldom heard’, ‘hard to reach’ or ‘marginalised’ groups in health and social care research is increasingly seen as important on scientific, policy and ethical grounds [1]. The under-representation of these groups in health research impacts the validity and generalisability of data, [2] the development of services and interventions that meet their needs, [3] resource allocation and access to it, [4] and health inequalities which are perpetuated as a result of this omission [5]. Reasons for this under-representation are numerous and multifactorial, and include the overarching design of a study, the assumptions of researchers, and ethical procedures [6]. The exclusion of some minority ethnic populations can also be due to concerns about language barriers [7].

South Asians are the largest minority ethnic group in the United Kingdom (UK). Although they constitute around 7.5% of the total British population, [8], they are under-represented in health research yet over-represented in the incidence of certain conditions such as cardiovascular disease, [9], type 2 diabetes, [10], depression, [11], and asthma [12]. Reasons for this under-representation in research can include the absence of people from these communities in the research team and hence the required language skills; and insufficient time and funding to facilitate recruitment and participation [5]. A study comparing United States and British researchers’ attitudes also found evidence of stereotyping among British researchers, including negative perceptions about the challenges of engaging with South Asian populations [12]. Barriers to recruiting South Asian participants to qualitative, quantitative and clinical trial research have been reported in several studies [3, 13] as well as difficulties in finding suitable staff with the relevant research, linguistic and cultural competencies [11, 14].

Although patient and public involvement (PPI) in research has developed considerably over the last 20 years, little is known about Black, Asian and Minority Ethnic (BAME) involvement or about the factors that influence this [15, 16]. With the growing requirement of patient involvement in research and the inclusion of diverse populations, [17] practical guidance on how to include, engage and conduct research with UK South Asian populations is essential if services are to be useful, relevant, ethical and impactful [18]. Methodological literature that offers such guidance, however, is limited, focusing mainly on the barriers and facilitators to recruitment, particularly to clinical trials. This is an understandable concern given that community-based randomised trials require much larger participant numbers and often greater participant commitment than qualitative research studies [19]. Such literature has focused on British South Asian recruitment to trials on asthma, [12] diabetes, [19] cancer, [20] cardiac rehabilitation, [21], and depression [11]. Much of it has emphasised the pivotal role of key members of the community, incentives and reciprocal benefits, and knowledge of South Asian cultures. Previous research has also noted the importance of building trust and personal relationships [22].

Methodological guidance for researchers working with UK South Asian communities, aside from recruitment, is much more limited. Although a range of different methods can be helpful when working with ‘seldom heard’ groups, [5] focus groups can be particularly useful to gain preliminary insights into a new exploratory study or area of research. The strengths of using focus groups with the general population, usually English-speaking groups in Western countries, have been discussed at length in previous literature and include the generation of a large amount of rich, interesting data in a relatively short timeframe [23], the observation of group interactions, and the co-construction of meaning [24]. Limitations include insufficient time for each participant to share all the details of their stories, the logistical challenges of organising focus groups, the time-consuming process of transcribing and analysing group discussions, and issues around ‘reliability’ [25].

Focus groups with British South Asian communities, though much less common, are also useful for the same reasons, as well as to gain an understanding of group norms, values and “community” responses to a topic [26]. Focus groups also enable people who are unwilling to be interviewed on their own to be involved in research, and do not exclude participants who cannot read or write [27]. Conducting focus groups with South Asian communities in Luton to elicit views on organ donation, Randhawa et al. highlight the importance of getting to know the local population before starting the study [14]. Drawing on their focus groups in three English cities, Culley et al. discuss the important role of community facilitators, with the relevant language and cultural knowledge, to access and recruit South Asian participants to discuss sensitive issues around infertility and assisted conception services [28].

In much of the qualitative and quantitative research with British South Asian populations, ‘community facilitators’ play a pivotal role because of their familiarity with South Asian languages, cultures and the local communities [29]. Community facilitators are often fluent in the required languages and are given brief training prior to conducting the research. Waheed et al. note the challenges of finding fully-trained researchers with all the required language skills, a skills gap, and the role of using community and social networks to find community facilitators or partners [11]. The role of community facilitators can include recruitment, data collection, translation and interpretation. We know little, however, about their involvement in research studies, and evolving roles, or about the optimal ways of working with them, particularly in contexts where language differences necessitate collaborative working with researchers. This paper seeks to address this gap.

## Methods

This article focuses on our experiences of conducting focus groups with South Asian communities in Leicester, which involved experienced community partners and researchers working closely together. We reflect on the factors that aided the successful delivery of the project, the challenges that we encountered, how we dealt with these, and recommendations.

### Ethical approval

Ethical approval was gained from the University of Oxford ‘Central University Research Ethics Committee’ (Ref R50751/RE001). All participants gave written informed consent to participate.

### The SuMMiT-D study (support through Mobile messaging and digital health Technology for Diabetes)

Our focus group study was part of a larger project that aimed to explore supporting people with type 2 diabetes (T2D) in effective use of their medicine through a system comprising mobile health technology integrated with clinical care [30]. A specific workpackage, titled ‘Exploring British South Asian people’s views of type 2 diabetes and support from brief mobile phone messages’, focussed exclusively on the perceptions and experiences of South Asian people with T2D. British South Asian populations include first, second and third generation people of Indian, Pakistani, Bangladeshi and Sri Lankan descent – communities that are diverse in terms of language, religion, migration history, country of birth, education, literacy, cultural norms, traditions, diet and food habits. UK South Asians are up to six times more likely to have T2D than the White British population, to develop diabetes at a younger age, and to experience complications [31]. Rates of type 2 diabetes also vary between communities, with British Bangladeshis suggested to have the highest risk [32]. Type 2 diabetes is predicted to increase in England by 47% by 2025 and will continue to have a significant effect on South Asian communities [10].

### Study design

Our exploratory focus groups aimed to investigate people’s challenges, support needs, and views on support for diabetes self-management through short text messages. Understanding the experiences of South Asian populations can help yield valuable insights into ‘self-management’, information and support needs, and appropriate interventions. We aimed to include a broad range of views, reflecting the heterogeneity within and across British South Asian communities.

In total we conducted eight focus groups with South Asian communities in Leicester between September 2017 and March 2018, a collaboration between the University of Oxford and the Centre for Black and Minority Ethnic Health (CBMEH), University of Leicester. A total of 67 participants (including 4 carers) were recruited from some of the largest South Asian communities in the UK: Indian Punjabi Sikh, Indian Gujarati Hindu, Pakistani Muslim, Bangladeshi Muslim, and Indian Gujarati Muslim. The eight focus groups were drawn from various communities in the following way: Punjabi Sikh men and women; Bangladeshi Muslim men; Pakistani Muslim men and women; Gujarati Hindu men and women; South Asian women; Bangladeshi Muslim women; Gujarati Muslim men; younger people aged 18–45. Groups were mixed and single-sex and ranged in size from *n* = 5 to *n* = 12 (Table 1).

**Table 1:** Focus group composition

Language/Cultural Group	Participants
1. Punjabi Sikh men and women	11
2. Bangladeshi Muslim men	11
3. Pakistani Muslim men and women	07
4. Gujarati Hindu men and women	08
5. South Asian women	12
6. Bangladeshi Muslim women	07
7. Gujarati Muslim men	05
8. Younger people aged 18–45	06
**TOTAL**	**67**

We aimed for a diverse sample in order to gain a broad range of experiences, including people of different age groups, generations, educational and occupational backgrounds, fluency in English, place of birth, and time since diagnosis (Table 2). The educational backgrounds of participants ranged considerably, from those who had minimal education in South Asia and/or Africa to those who had obtained a doctorate from a UK university. We did not have the educational details of all participants and so this information is excluded from Table 2. Literacy levels and fluency in English also varied. Participants were recruited by NM from community centres, places of worship, and a South Asian women’s centre. Discussions were held in community centres that participants were familiar with and could easily access. One focus group was held in a centre for South Asian women. Each group met once. A topic guide was used, informed by the medical and social science literature on British South Asian experiences of diabetes and in discussion with the wider research team and CBMEH. Topics raised by participants in the discussions included diet, healthcare, medication, community events, family support and information. The groups lasted between 1.5 and 2 h.

**Table 2:** Demographics of focus group participants

	Male	Female	Age range	Country of birth
**Punjabi Sikh**	5	6	47–78	India (11)
**Bangladeshi Muslim**	11	0	41–81	Bangladesh (10) UK (1)
**Pakistani Muslim**	3	4	39–66	Pakistan (3) India (1) Bangladesh (1) Malawi (1) Mozambique (1)
**Gujarati Hindu**	4	4	56–84	India (4) Kenya (2) Uganda (1) Trinidad (1)
**South Asian**	0	12	18–71	Bangladesh (3) Pakistan (3) India (2) Sri Lanka (1) Uganda (1) Malawi (1) UK (1)
**Bangladeshi Muslim**	0	7	34–45	Bangladesh (7)
**Gujarati Muslim**	5	0	50–75	India (4) Malawi (1)
**Younger people**	1	5	28–47	Bangladesh (6)

## Results

### Reflections on challenges and opportunities of conducting focus groups

Findings from this study are being reported separately [33, 34]. Here we reflect on the challenges and opportunities that we encountered when conducting focus groups with South Asian communities in Leicester. We have grouped our experiences into four categories: co-working with community partners; linguistic competency; cultural competency and awareness; and reflexivity, power and acknowledgement.

### Co-working with community partners

Key to the success of our focus groups was collaborative team work, which involved experienced people with different kinds of knowledge on diabetes and British South Asian cultures working together.

#### Team experience and roles

AF, Professor of General Practice and a GP, was Principal Investigator of the study. He obtained funding for the project, contributed to the study design, and advised on the topic guide. The core focus group research team consisted of SP, NM and EA. SP was a senior qualitative researcher (anthropologist) at the University of Oxford. She had over 17 years of postdoctoral research experience, including qualitative interviews with South Asian participants. NM was a project support worker at the University of Leicester, where she had been working at the CBMEH for 4 years. She had also worked for over 10 years as a project worker at the Bangladesh Youth and Cultural Shomiti (BYCS), a community organisation in Leicester that provides advice on education, training and employment. EA was a patient partner, a project officer at the BYCS, and a community champion with the CBMEH. He had been living with T2D since 2010.

All three core team members were involved in data collection because of their knowledge and experience with different British South Asian cultures, languages and communities. NM was responsible for recruitment as her role at the CBMEH had involved recruiting people from local communities to research studies and engagement activities. This had included building relationships and links with local organisations, faith leaders, community centre staff and other ‘gatekeepers’. NM was also fluent in spoken Bengali, Sylheti, Hindi and Urdu.

EA had facilitated focus groups in Bengali and the Sylheti dialect for previous CBMEH projects and was responsible for conducting a focus group with Bangladeshi Muslim men and one with Bangladeshi Muslim women. Having EA, a member of the Bangladeshi community and a patient partner, as a facilitator of the two Bangladeshi Muslim focus groups had several advantages. Some participants knew him already and so trust and relationships had already been established. Participants might also have felt more comfortable discussing their views with someone from their own community or someone that they knew and trusted. Mehta notes that familiarity with moderators, however, can also hinder disclosure, [35] and this may be particularly pertinent when the discussion topic is very sensitive. Although there can be issues with using a ‘non-researcher’ in terms of the depth of probing that they will undertake, [36] having NM and SP present at both focus groups helped ensure that topics were explored in detail. Moreover, being a patient partner EA was able to bring his own experiences into the discussions to prompt conversation, particularly on family support and the impact of T2D on other family members.

SP was lead researcher, had overall responsibility for the study, and could communicate in Punjabi, Urdu and Hindi. Because SP, NM and EA were familiar with focus group research, the discussion topic, and South Asian languages and cultures, extra training had not been required. Specific issues can relate to the brief training of bilingual staff in research methods and the discussion topic, including the potential impacts of this on probing and data richness. Combining their experience and language skills, the three core team members were able to conduct focus groups in the preferred language of participants. The team was supported by bilingual assistants recruited by the CBMEH to help with form-filling at the start of some of the focus groups, which took participants around 20 minutes.

Having an experienced team familiar with the heterogeneity within and across British South Asian communities helped ensure that our sample reflected this diversity. Where this was most noticeable was in the inclusion of people who spoke only in a South Asian language. On one occasion, a community centre recruiter felt that participants who were fluent in English and familiar with using digital devices would be the most suitable participants from their community centre. Discussing this with them, NM clarified the reasons for including participants who were not fluent in English or familiar with technology, and ensured that the group would include younger and older participants who met this criteria. NM’s experience and familiarity with the local communities helped us to recruit a broad range of participants. She was, however, often reliant on community centre workers and other gatekeepers to inform potential participants of the study.

As an experienced team, as well as trying to ensure that we recruited a diverse sample, we also encountered other challenges. Although NM and the CBMEH had good links and contacts with local organisations, which helped in terms of access, recruitment and the project running to schedule, recruitment was still sometimes difficult. The Gujarati Muslim men’s group, for example, had to be rescheduled due to a lack of participants. Some groups were small but, when NM contacted recruiters on several further occasions, the group became larger than expected. Both NM and SP were reluctant to turn people away, though, preferring instead to be flexible on the day. The smaller groups were generally easier to facilitate and enabled more contributions from each participant.

#### Collaborative working from research design to dissemination

Working collaboratively was integral throughout the process from research design to dissemination. SP began working with NM in November 2016, 10 months before any of the focus groups had been set up, and more closely during the data collection phase. Co-working included numerous telephone, email and face-to-face discussions about the study methods, ethics forms, recruitment, the role of languages, staff roles, the topic guide, whether and when to have single-sex or mixed groups, the translation of study materials into South Asian languages, and dissemination. Table 3 highlights some of the key considerations we encountered. SP, NM and AE also worked together to disseminate the research findings at a PPI workshop held in Leicester in June 2018, where participants discussed their views on future research too [37]. Workshop participants included community centre members, faith leaders, healthcare professionals, focus group participants and researchers. Although patient involvement in health research is often limited to the early stages of research, such as advising on research questions and design, [38] in our study EA was involved in data collection and dissemination too.

**Table 3:** Key considerations from our focus group study

**Study design**	• Which communities need to be included in the research and why? • How heterogeneous are these groups (e.g. in terms of cultures, religions, languages, age, education, generation, migration history)? • Which organisations / individuals can advise throughout the study on ethics, access, recruitment, methods, topic guide and dissemination?
**Research team**	• Composition of team, including researchers, community and patient partners, and support staff with relevant language and cultural competency skills • Gender - will we need male and female researchers and community facilitators? • Any training needs?
**Institutional procedures**	• Check institutional procedures including policies on ethics, translation of materials, and payments to participants
**Costs**	• Staff, including those with linguistic and cultural skills • Participant incentives • Translation and transcription • Room hire • Transport and childcare costs • Sufficient time to build relationships, recruit, translate transcripts
**Building relationships**	• Contact and visit relevant organisations, support groups, individuals, community centres and places of worship to start building trust and relationships as early as possible • Involve and inform relevant organisations / individuals throughout the study • Invite community organisations and partners to participate in dissemination activities
**Ethics and informed consent**	• Verbal, audio or written consent? • Do forms need to be translated into South Asian languages and piloted before use? • Forms need to be as clear, accessible and as short as possible • Who will explain and take consent?
**Recruitment**	• Use a range of strategies and sources. This could include local and national Asian TV and radio stations • Does the sample reflect the diversity within and across communities?
**Venue**	• How easy is the venue for participants to access? • Transport costs to and from venue
**Focus groups**	• How many focus groups will need to be conducted? • Have all relevant communities been included? • Size of groups – smaller groups of around 5–7 participants worked best • Timing – avoid religious festivities and Ramadan; be aware of childcare responsibilities and shift work • Mixed or single-sex groups? • Older and younger participants together or separate? • How will confidentiality be discussed and ensured? • Flexibility - participants may arrive late or leave early due to other responsibilities. Be prepared that there may be more or fewer participants on the day than expected
**Translation and transcription**	• Who will do this? • If using community facilitators, what are the strengths and limitations of this? • If using a professional translation agency, how long will they take and how much will it cost? Strengths and limitations?
**Analysis / interpretations**	• Involve staff with cultural and linguistic knowledge and skills • Consider presenting findings to participants for feedback
**Reflexivity, power and acknowledgement**	• How are the data shaped by various stakeholders? • What can we do to minimise power differentials? • How will all contributors be acknowledged?
**Dissemination**	• How will the findings be communicated to stakeholders, including patients and public? • How can community and patient partners be involved in dissemination? • Can we make use of local and national Asian TV and radio stations as avenues for communicating the findings? • Invite community and patient partners to co-author papers and present at conferences

#### Being present at every focus group

SP and NM were present at every focus group regardless of whether they were fluent in the language that the discussion would be conducted in. SP, for example, was not fluent in Bengali but could understand some, and some words and sentences were spoken by participants in English. Being present during discussions enabled SP and NM to observe group dynamics, to answer any questions that participants might have, to be available to help with form-filling, to prompt on issues that were covered very briefly, to observe similarities and differences between all eight focus groups, and to help contribute to data quality and richness. Being present was particularly beneficial when topics were not covered in depth or when demographic information seemed difficult to make sense of. In the Bengali Muslim women’s group, for example, SP noticed on some of the demographic forms that participants had migrated to the UK from Bangladesh via Italy. Querying this prompted a comparison of a participant’s experience of diabetes healthcare in Italy and the UK. On another occasion, SP was able to observe in the Bangladeshi Muslim men’s group that two participants in their early forties were unable to speak, read or write in English and needed help with form-filling. This information is impossible to gauge if researchers only receive completed forms filled out in English by facilitators.

NM and SP were also able to observe the dynamics between different age groups, generations and genders, and to discuss their reflections afterwards. Being present enabled them to discuss how they would work together before each focus group, how they felt the discussion had gone afterwards, whether any changes were needed to subsequent groups in terms of the format, sampling and recruitment, and to reflect on their individual experiences. Both kept notes of their reflections which they discussed on an ongoing basis as well as before drafting the paper. This helped ensure that any issues or challenges were dealt with as soon as they arose.

### Linguistic competency

Linguistic competency – i.e., fluency in the preferred language of participants – was crucial to facilitating recruitment, data collection, and the translation and transcription of focus group discussions. Having three people in the research team who could communicate in the various languages spoken by participants was also important in terms of building relationships and rapport. The focus group discussions were conducted in Punjabi, Bengali, Sylheti, Urdu, Hindi and English. Although some of the discussions included a mix of English and a South Asian language or dialect, two discussions were conducted almost completely in English (the Gujarati Hindu and Pakistani Muslim groups) – a decision made by participants at the start of the focus group. This may have been influenced by the overall education of participants and because SP and NM were both second generation co-facilitators. The discussions included Gujarati and Urdu words and sentences, too, including conversations about South Asian foods which were almost always discussed using the South Asian food names. The younger people’s focus group was also conducted in English. One participant whose main language was Bengali expressed her views through NM who translated in real time.

From the outset of our exploratory study we agreed that, because NM and SP would be working together throughout the project, the patient information sheet, consent form and a brief demographic information form would be available in English only and that all the forms would be discussed at the start of the focus group, one-to-one with participants where required. The aim of the focus groups was also explained to participants at the start of each discussion and any questions answered. Some participants had been given hard copies of the forms prior to the focus group but most had not read them, preferring instead to have them explained verbally. Previous research with South Asian communities also highlights that participants often do not read the paperwork, preferring to have it explained verbally whether they are literate or not [39]. Waheed et al. also note that some South Asian people speak a language but are unable to read it. They, as well as other researchers, have discussed at length issues around the translation of forms, having forms in English and the relevant South Asian languages, and the need to pilot these [11]. These are important considerations that also add to the time and costs of a project. In our exploratory project, because of the vast collective experience of the research team, as well as time and resource considerations, we felt that using English language forms would be acceptable (and this proved to be the case).

All focus group discussions were recorded. The Punjabi, South Asian women’s, and Gujarati Muslim men’s groups were translated and transcribed by SP. Two discussions that were mostly conducted in English were transcribed by a professional transcriber and checked for accuracy by SP, who inserted the South Asian words and sentences. Although the discussions were mostly in English, checking transcripts can still be very time-consuming. A range of different accents and volumes, participants occasionally speaking over one another, and background noise can all contribute to this. Being present at the focus group helps and provides the broader context within which views are expressed. Following University of Oxford policy, an approved translation agency was used to translate and transcribe the Bangladeshi Muslim men’s and women’s focus groups. Community facilitators often undertake translation and transcription but institutional procedures prevented us from being able to do this on this occasion. This is a point worth considering in our future work in terms of the strengths and limitations of using community partners rather than professional translators, including costs. Although facilitators are present at focus groups and may be familiar with translating discussions, Sharp et al. caution that they may produce less detailed translations than professional translators [40].

### Cultural competency and awareness

Culley et al. state that linguistic competency alone does not ensure good rapport between facilitators and participants, and that facilitators need to be familiar with the culture of the group too [28]. Waheed et al. note the importance of demonstrating religious knowledge and sensitivity, and that cultural understanding gives research participants confidence that they will be understood [11]. Participant feedback confirms that this is often a key consideration when agreeing to take part [41]. While ‘religion’ and ‘culture’ are huge topics well beyond the scope of this paper, when NM and SP discussed their reflections and observations, certain topics were significant. They were conscious, for example, of ensuring that focus groups were held on days and times that did not clash with Sikh, Hindu or Muslim religious festivities, prayer times, with Ramadan, or with childcare responsibilities. Familiarity with cultural norms also included an awareness and respect in terms of age, generation, gendered roles, and dress code (see also Zubair et al. 2012 and Waheed et al. 2015). Participants were addressed using kinship terms (such as aunty, uncle, brother, sister) rather than their names. Zubair et al. also used particular forms of addressing their participants as this was more culturally appropriate and demonstrated cultural awareness and respect [42].

SP and NM learnt from one another as well as from the academic literature to gain a deeper understanding of the different UK South Asian communities. They regularly discussed their professional and personal knowledge of these populations as well as the diversity within and across groups. Decisions about whether to have single-sex or mixed groups included considerations around cultural and religious norms and expectations, particularly when men and women may feel more comfortable in single-sex groups. Familiarity with the different South Asian cultures and religions also helped when devising the topic guide and when prompting during discussions. This was particularly pertinent in discussions around the challenges of adhering to a healthy diet and to medications as prescribed when attending family and community events, travelling to South Asia, and when fasting.

A key topic in all of the focus groups was diet and South Asian foods, including discussions around different types of chapatti flour, herbs and spices used in cooking, meat versus vegetarian diets, and foods that were perceived to be helpful in managing T2D. The South Asian names of foods were used even when the rest of the discussion was in English, and a shared understanding of these foods was often assumed by participants. One of the advantages of this was the depth of discussion it enabled, without the need for lengthy introductory explanations or descriptions. Had we been unfamiliar with these foods, we would have had to prompt for clarification which may have hindered a deeper discussion.

### Reflexivity, power and acknowledgement

Previous research encourages using reflexive approaches that consider how the similarities and differences between researchers and participants shape the research process and knowledge generated [43]. Zubair et al. note how the young Pakistani Muslim researcher in their study, on older Pakistani Muslim people’s experiences of ageing, was simultaneously an ‘insider’ in relation to some aspects of her identity and an ‘outsider’ in others. Gender, age and ethnicity intersected with social class and generational difference [42]. SP and NM regularly reflected on their various identities as female, Indian, Bangladeshi, Asian, British and profesional and the potential impact of these on recruitment, the focus group discussions, data analysis and interpretation. NM, for example, felt that recruiting the Bangladeshi groups was easier for her than some of the other groups. Familiarity with the language, culture, religion and local organisations helped, and she was seen as an ‘insider’. Perceptions of ‘outsider’ status may have made recruiting participants to some of the other focus groups more difficult.

Reflexive methodologies encourage researchers to consider power relationships between themselves and their participants. Previous research has drawn attention to issues around the power differentials when White British researchers conduct research with BAME research participants [44] as well as to the complex power relations when South Asian researchers conduct research within Asian communities, including the interplay of power with age, ethnicity, gender, education and class [42]. Power differentials were complex in our study, too, and NM and SP regularly reflected on the intersections of age, gender and education in our focus groups where they were female, and often younger than their participants and addressed them as aunty and uncle; and where participants were often curious about SP’s background as a researcher at Oxford and someone from outside Leicester, sometimes noting similarities and differences across South Asian communities, generations, and in migratory histories.

Power differentials are also discussed in the growing literature on the coproduction of research where, in principle, power and responsibility are shared between researchers, health professionals and the public, including the generation of knowledge. Different kinds of knowledge (experiential, clinical and academic) are valued as equal, as are the contributions of each team member [45]. Although our study was not coproduced, each member of the core team had different kinds of knowledge, skills and roles, all of which contributed to the generation and richness of the data and the successful delivery of the project. Although community and patient partners are often mainly involved in the early stages of research, NM and EA were involved in conducting research, interpreting the data and dissemination too, including co-authoring papers (in progress) and subsequent grant applications. Best practice in patient engagement research suggests providing recognition for patient contributions, [46] which was important to us as a team given the valuable and unique contributions of each team member. Thus, EA was acknowledged for all of his work in the focus group study and at the PPI workshop. NM was also acknowledged for her support with recruitment, setting up and co-facilitating the focus groups, and jointly organising the workshop. Partnerships are ongoing, including involvement in funding applications.

## Discussion

Although British South Asian communities are under-represented in health research, our study suggests ways to involve and conduct focus groups with these populations. Collaborating with local community organisations and experts is key to this, as well as having an experienced team. Waheed et al. recommend inviting minority ethnic community facilitators and researchers to be co-investigators on studies because they can contribute cross-cultural methodological skills and knowledge [11]. Halcomb et al. believe that the success of focus groups in cross-cultural contexts is dependent upon the cultural competence of the research team [26]. In our study, having an experienced researcher, project support worker and patient partner combining their different kinds of professional and personal knowledge of diabetes, and of South Asian communities, languages and cultures, was an optimal way of working.

Another important consideration when planning to conduct research with South Asian communities is the linguistic and cultural competencies of the core team, any training that might be required – including in research methods, the discussion topic, and cultural competency – and the limitations of brief training. Working with relevant organisations and having an experienced team fluent in the required languages is key when accessing communities, recruiting participants, and establishing relationships. It can also instil greater confidence in the study [47]. Using community centre staff and other organisations to recruit participants can, however, be challenging and requires clear and frequent communication. They may, for example, be unfamiliar with research and select participants on the basis of specific criteria such as fluency in written and spoken English. Discussions about the study aims, methods and the importance of a diverse sample can help. Building trust and relationships with local communities and organisations can take time. It needs to start before the research design phase, and to be accounted for in terms of study time and costs. Organisations such as the CBMEH provide advice and guidance on language and cultural issues and designing studies with BAME communities.

The inclusion of ‘seldom heard’ views is essential if health research and interventions are to be relevant to these groups. Many of the factors that we had to consider in our study are relevant to researchers planning to conduct research with other seldom heard groups. This includes building relationships, rapport and trust with relevant organisations and people, involving community partners and experienced researchers as part of the core research team as early as possible, reflecting on how the data are shaped by different stakeholders, power differentials and how to share power, and the importance of acknowledging the contributions of community and patient partners.

Focus groups can be a useful method to explore a new area of research or topic with British South Asian communities. Setting up and conducting these groups has some similarities with those conducted with the general population. They can, for example, be challenging to schedule and moderate, and analysing large amounts of data can be time consuming [25]. There are also some considerations, however, that are specific to UK South Asian communities such as the linguistic and cultural competencies of researchers, access and recruitment, and translation, transcription and interpretation.

Having an experienced team working collaboratively saved time and the project ran to schedule. Cost implications also need to be considered when planning projects, including the costs of employing an experienced researcher, community and patient partners, support staff, translation and transcription, incentives to participants, venue hire, and transport and childcare costs where relevant.

### Strengths and limitations

This exploratory focus group study has several limitations, some of which are common to focus groups more generally. For example, the groups varied in size. Some were small and particularly difficult to recruit. All were held during the day. Evening focus groups might have encouraged more professional and younger people to attend. The participation of more second generation and some third generation participants may have helped generate further themes. A larger sample could be considered in future research to include more second and third generation participants and professionals. Focussing only on diabetes may present limitations too. Discussing more sensitive topics, for example, may introduce other considerations not discussed here. However, we did recruit a diverse sample of people who helped generate rich data. Participants seemed engaged and enthusiastic, and some volunteered to be involved in future research. Further, the themes discussed in this article have also been important in our previous studies and engagement activities with British South Asian participants.

## Conclusions

Focus groups with UK South Asian communities can be a useful way of exploring new topics and involving ‘seldom heard’ views. While a useful method, they are only one way of exploring a research topic and provide an insight into context-specific attitudes and views. Future research should explore British South Asian participants’ views on how they would like to be involved in research, including new methods of collecting data and coproducing research. While it is important to develop deeper understandings of South Asian experiences of diabetes, it is equally important to be aware of over-emphasising cultural differences. Differences and similarities exist within and across South Asian groups, and with the general population, and future research should help shed further light on these.

## Data Availability

All data are held at the University of Oxford. We do not have ethical approval to share the data.

## Abbreviations

PPI: Patient and Public Involvement
BAME: Black, Asian and Minority Ethnic
CBMEH: Centre for Black and Minority Ethnic Health
BYCS: Bangladesh Youth and Cultural Shomiti
